# Identifying MASLD Patients with MASH or Significant Fibrosis: The Role of Lower ALT Thresholds

**DOI:** 10.3390/jcm15187212

**Published:** 2026-09-17

**Authors:** Ying Ma, Hang Yang, Yuanyuan Zhao, Ping Han, Yuying Jin, Lili Zhao, Jie Liu, Jia Li

**Affiliations:** 1 Department of Gastroenterology and Hepatology, Tianjin Second People’s Hospital, Tianjin 300192, China; ma.ying0403@163.com (Y.M.); hanghm@foxmail.com (H.Y.); zhll_stone2013@tmu.edu.cn (L.Z.); 2 The Second People’s Hospital Affiliated to Tianjin Medical University, Tianjin Medical University, Tianjin 300203, China; zyy_001010@163.com (Y.Z.); 17836215668@163.com (Y.J.); 3 Department of Hepatology and Gastroenterology, Tianjin First Central Hospital, Tianjin 300190, China; 13652139315@163.com

**Keywords:** metabolic dysfunction-associated steatotic liver disease, fibrosis, pathology, missed diagnosis

## Abstract

**Background:** Serum ALT is widely used to assess liver inflammation and fibrosis, yet metabolic steatohepatitis (MASH) and significant fibrosis can still occur in metabolic dysfunction-associated steatotic liver disease (MASLD) patients with normal ALT. However, liver histology in patients with ALT below the cutoffs remains poorly characterized. This study aimed to compare the diagnostic performance of conventional and lower ALT thresholds for assessing MASH and significant fibrosis, using liver biopsy as the reference standard. **Materials:** This retrospective cross-sectional, two-center study included 404 patients with MASLD, without concurrent viral hepatitis, who underwent liver biopsy at two hospitals in Tianjin between October 2012 and October 2024. The assessment of inflammation and fibrosis was based on liver biopsy histology. Patients were stratified using both conventional and lower ALT thresholds (30 U/L for men, 19 U/L for women). **Results:** Among the 404 patients, 70.3% had MASH and 67.1% had significant fibrosis. Of those with conventionally normal ALT levels (*n* = 130), the prevalence of MASH and significant fibrosis was 49.23% and 50.00%, respectively. Using a lower ALT threshold, patients with ALT between the conventional and lower thresholds had significantly higher rates of significant fibrosis compared to those with ALT below the lower threshold (56.18% vs. 36.59%, *p* = 0.002). The incidence of MASH also showed a decreasing trend in the lower ALT group (53.93% vs. 39.02%, *p* = 0.082). Multivariate analysis showed that the application of a lower ALT ULN was independently associated with MASH (*OR* = 5.188, 95% *CI*: 1.494–18.018, *p* < 0.001). **Conclusions:** The study evaluated the association between liver histology and ALT under alternative externally proposed ALT thresholds. The lower ALT threshold was independently associated with MASH, but its diagnostic performance was limited; further prospective evaluation is warranted.

## 1. Introduction

Metabolic dysfunction-associated steatotic liver disease (MASLD) represents a growing global health burden, encompassing a disease spectrum that ranges from simple steatosis to metabolic steatohepatitis (MASH), cirrhosis, and hepatocellular carcinoma (HCC) [1,2,3,4]. Despite its high prevalence, a substantial number of cases remain undiagnosed and untreated [5]. Notably, a significant proportion of MASLD patients develop MASH and significant fibrosis even in the setting of normal serum alanine aminotransferase (ALT) levels [6,7]. Although liver biopsy remains the gold standard for diagnosing MASH, its use is limited in clinical practice, particularly among patients with normal ALT [8,9]. With advances in the management of viral hepatitis, MASH is projected to become the leading cause of cirrhosis worldwide [2].

Interestingly, a similar diagnostic challenge exists in chronic hepatitis B (CHB), where patients with normal ALT may still exhibit substantial histological activity and fibrosis [10,11]. This has prompted experts in East Asia to propose lower ALT upper limits (30 U/L for men and 19 U/L for women) [12], as high-normal ALT levels are associated with increased risks of cirrhosis and liver-related mortality [11,12,13,14]. Studies applying these lower thresholds in CHB have shown that patients with ALT above 1× the revised ULN have a considerably higher prevalence of advanced histological injury than those below the threshold [12,13,14].

These findings demonstrate that in CHB, even mild ALT elevations within the conventional normal range are associated with a stepwise increase in histological risk. Given the parallels between MASLD and CHB regarding the insensitivity of conventional ALT thresholds, we aimed to evaluate the application of lower ALT thresholds in MASLD. Specifically, this study used liver biopsy as the gold standard to assess hepatic inflammation and fibrosis, compared the agreement of conventional and lower ALT thresholds with histological findings, and combined non-invasive methods to evaluate their diagnostic performance.

## 2. Materials and Methods

### 2.1. Study Population

We reviewed the medical records of adult patients with MASLD who underwent liver biopsy between October 2012 and October 2024 at Tianjin Second People’s Hospital and Tianjin First Central Hospital. MASLD was defined according to The Asian Pacific Association for the Study of the Liver clinical practice guidelines [15] as the presence of hepatic steatosis detected by liver biopsy, imaging, or non-invasive biomarkers, together with at least one of the following: overweight/obesity, type 2 diabetes mellitus, or clinical evidence of metabolic dysfunction in lean individuals. Patients with concurrent viral hepatitis, hepatocellular carcinoma, significant alcohol consumption (defined as ongoing or recent intake >210 g/week for men and >140 g/week for women), drug-induced liver injury, autoimmune hepatitis, primary biliary cholangitis, or other genetic and metabolic liver diseases were excluded. The presence of MASLD and MASH was assessed by physicians at the two study centers, and any disagreement was resolved by a third physician. In this study, MASH was defined histologically as severe inflammation characterized by steatosis, lobular inflammation, and hepatocyte ballooning, with an NAFLD activity score (NAS) >4 used as the assessment indicator. Significant fibrosis was defined as fibrosis stage ≥F2 on liver biopsy histology. Finally, a total of 404 MASLD patients were included in this cross-sectional analysis. The study protocol conforms to the ethical guidelines of the 1975 Declaration of Helsinki (as revised in Brazil 2013) and Istanbul as reflected in a priori approval by the institution’s human research committee. The protocols and informed consent forms were approved by the Ethics Committee of Tianjin Second People’s Hospital (Number 2021-55). Informed consent was obtained from each patient included in the study.

### 2.2. General Characteristics and Laboratory Test Results

This study examined the medical records of all 404 enrolled patients with MASLD. Data on demographic characteristics (gender and age), laboratory tests (complete blood count, biochemistry, and immunology), FibroScan parameters [controlled attenuation parameter (CAP) and liver stiffness measurement (LSM)], and histopathological assessments from liver biopsy were obtained for analysis. Blood samples were collected from all participants within one week prior to the liver biopsy procedure. Platelet (PLT) counts from complete blood analysis were determined using a Sysmex XN-2000 hematology analyzer (Sysmex Corporation, Kobe, Japan). Serum levels of alanine aminotransferase (ALT), aspartate aminotransferase (AST), gamma-glutamyl transferase (GGT), albumin (ALB), fasting blood glucose (FBG), triglyceride (TG), and low-density lipoprotein cholesterol (LDL-C) were measured with a Hitachi 7180 Automatic Biochemical Analyzer (Hitachi Limited, Tokyo, Japan). Ferritin detection was performed on a Microlab STAR IVD ELISA workstation (Hamilton Limited, Bonaduz, Switzerland). CAP and LSM were assessed using a FibroScan 502 Touch device (Echosens Limited, Paris, France). The conventional upper limit of normal (ULN) for ALT was defined as <50 U/L for men and <40 U/L for women [16]. A lower ULN was applied as 30 IU/L for men and 19 IU/L for women [12].

Among patients with conventionally normal ALT levels (<50 U/L for men and <40 U/L for women), those with ALT values between the lower and conventional ULNs (30–50 U/L for men and 19–40 U/L for women) were categorized as the high-threshold normal group. Patients with ALT levels below the lower ULN (<30 U/L for men and <19 U/L for women) were designated as the low-threshold normal group. Liver biopsy procurement aimed for one or two specimens containing a minimum of 10 portal tracts. All histological samples were evaluated independently by two experienced liver pathologists who were blinded to the clinical data. Liver fibrosis was staged from 0 to 4, with stages ≥F2 considered significant fibrosis [2]. The aspartate transaminase-to-platelet ratio index (APRI) and the fibrosis-4 index (FIB-4), established models for fibrosis prediction, were calculated [2]. For the detection of MASH, a CAP value >301 dB/m was used. The thresholds for identifying significant fibrosis were set at APRI >1.5, FIB-4 >3.25, and LSM >7.0 kPa [17,18]. Patients were stratified by age using a cutoff of 50 years and compared across genders [19,20,21].

### 2.3. Statistical Analysis

The Kolmogorov–Smirnov test or Shapiro–Wilk test was used to test whether continuous variables conform to normal distribution. Continuous variables with a normal distribution were expressed as mean and standard deviation, while continuous variables with a non-normal distribution were expressed as median (interquartile range). Categorical variables were expressed as frequencies and percentages. Student’s *t* test or the Mann–Whitney U test were used for the comparison between groups. Multiple logistic regression analysis was used to explore the association of clinical variables with MASH or significant fibrosis. *p* < 0.050 was considered as statistically significant. Statistical analyses were conducted using Excel and SPSS 27.0.

## 3. Results

### 3.1. General Characteristics of the Patients with MASLD by Conventional ALT Upper Limit of Normal

We stratified the entire cohort (*n* = 404) according to the conventional ALT upper limit of normal (50 U/L for men, 40 U/L for women). Compared with patients with ALT at or above this threshold (*n* = 274), those with ALT below the threshold (*n* = 130) were older (median 51 vs. 39 years, *p* < 0.001) and more likely to be female (59.3% vs. 43.8%, *p* = 0.004) and had a higher prevalence of type 2 diabetes mellitus (40.8% vs. 29.2%, *p* = 0.023). They also had significantly lower levels of TG, LDL-C, CAP, AST, GGT, albumin, PLT, and ferritin (all *p* < 0.050). Histologically, patients with ALT below the threshold also exhibited less severe steatosis, lobular inflammation, and hepatocyte ballooning (all *p* < 0.001). Although patients with ALT below the conventional upper limit of normal had lower rates of MASH and significant fibrosis, a substantial proportion (49.23% and 50.00%, respectively) still had histologically advanced disease (Table 1).

### 3.2. Characteristics of MASLD Patients with Conventionally Normal ALT, Stratified by a Lower ALT ULN

When grouping was based on the lower ALT threshold (30 U/L for males and 19 U/L for females), patients with ALT at or above this threshold had significantly higher levels of ferritin, CAP, and APRI than those with ALT below the lower threshold (Table 2). No significant differences were observed between the two groups in age, sex, BMI, type 2 diabetes, GGT, PLT, FBG, TG, LDL-C, or FIB-4. Histologically, although the prevalence of MASH did not differ significantly between the two groups, the proportion of significant fibrosis was significantly higher in patients with ALT at or above the lower threshold than in those below (56.18% vs. 36.59%, *p* = 0.002). MASH remained present in 39.02% of patients with ALT below the lower threshold.

### 3.3. General Characteristics of MASLD Patients with Conventionally Normal ALT, Stratified MASH or Significant Fibrosis

When stratified by the presence of MASH or significant fibrosis confirmed by liver biopsy, intergroup comparisons revealed that patients with MASH or significant fibrosis were older and had significantly higher levels of BMI, ALT, FBG, LSM, CAP, APRI, and FIB-4, along with significantly lower PLT counts, compared to those without these conditions (Table 3). No significant differences were observed between the two groups in terms of gender distribution, type 2 diabetes, GGT, TG, LDL-C, or ferritin levels.

### 3.4. Histological Differences in MASLD Patients with Conventionally Normal ALT Stratified by Age and Gender

Further analysis was performed to evaluate histopathological differences based on age and gender using the lower ALT ULN. When stratified by age (using 50 years as the cutoff), no significant differences were observed in the prevalence of MASH or significant fibrosis between groups with ALT levels above or below the lower ALT ULN, either in the older or younger subgroup (Table 4).

In the gender-based analysis, among males, there was no significant difference in the prevalence of MASH between those with ALT above and below the lower ALT ULN. However, a significantly higher proportion of significant fibrosis was observed in males with elevated ALT (57.58% vs. 40.00%, *p* = 0.03, Table 5). In contrast, among females, significant differences were found in both the prevalence of MASH (60.71% vs. 28.57%, *p* = 0.001) and significant fibrosis (55.36% vs. 33.33%, *p* = 0.021) between those with ALT above and below the threshold.

### 3.5. Independent Risk Factors for MASH and Significant Fibrosis in Patients with Conventionally Normal ALT

Multivariate analysis showed that the lower ALT ULN was independently associated with MASH (*OR* = 5.188, 95% *CI*: 1.494–18.018, *p* < 0.001). However, age >50 years was not significantly associated with either MASH (*OR* = 1.743, 95% *CI*: 0.549–5.530, *p* = 0.378) or significant fibrosis (*OR* = 1.043, 95% *CI*: 0.391–2.780, *p* = 0.933). Similarly, sex did not show a significant association with either outcome (*p* = 0.346 for MASH, and *p* = 0.284 for significant fibrosis, Table 6).

To further explore the clinical utility of these markers, we evaluated the diagnostic performance of ALT, CAP, LSM, FIB-4, and APRI for detecting MASH or significant fibrosis. Among the 130 patients with conventionally normal ALT, applying the lower ALT threshold (≥30 U/L for men, ≥19 U/L for women) yielded an AUC of 0.649 for MASH, with a sensitivity of 84.4%, specificity of 45.5%, positive predictive value of 60.0%, and negative predictive value of 75.0%. For significant fibrosis, the AUC was 0.623, with a sensitivity of 84.4%, specificity of 83.3%, positive predictive value of 83.1%, and negative predictive value of 84.6%. The ROC curves for all evaluated markers are presented in Figure S1.

## 4. Discussion

Elevated aminotransferase levels often indicate liver inflammation and the development of fibrosis. However, in clinical practice, a considerable proportion of patients with ALT below the conventional threshold still have liver inflammation and advanced fibrosis, and the use of traditional ALT thresholds often leads to missed diagnosis [22]. Unlike studies that use non-invasively defined endpoints to classify MASH or significant fibrosis, our investigation is based on a well-characterized cohort of 404 MASLD patients, each with complete liver histopathology allowing definitive assessment of MASH or significant fibrosis. This universally histology-verified design provides a more accurate and direct assessment of disease activity. When participants were initially stratified using standard ALT cutoffs, those with elevated ALT were not only younger but also exhibited more severe histologic features, including intensified inflammation, fibrosis, and dysregulated lipid metabolism. Crucially, we identified a substantial subset of patients with equally significant histological disease despite ALT levels within the conventional normal range. This finding, consistent with reports such as that by Kawanaka M et al. [23], highlights a critical limitation of the current ALT upper limit of normal. Revising these cutoffs may help to more accurately capture inflammatory and fibrotic activity in MASLD and, in turn, to reduce diagnostic oversight and ensure timely clinical intervention.

In this biopsy-confirmed MASLD cohort, nearly half of patients with ALT within the conventional normal range still had MASH (49.23%) or significant fibrosis (50.00%). Applying the lower ALT thresholds previously proposed for chronic hepatitis B (30 U/L for men and 19 U/L for women) [11,12,13,14], the rate of significant fibrosis was significantly higher in patients with ALT above these lower thresholds than in those below (56.18% vs. 36.59%, *p* = 0.002); a similar trend was observed for MASH, though it did not reach statistical significance (53.93% vs. 39.02%, *p* = 0.082).

We further analyzed sex-related differences in these associations. The rates of both MASH and significant fibrosis were significantly higher among female patients with ALT levels between 19 and 40 U/L than in those with ALT below 19 U/L (MASH: 60.71% vs. 28.57%; fibrosis: 55.36% vs. 33.33%). This finding suggests that, as in chronic hepatitis B, lower ALT thresholds may also help identify a subset of MASLD patients with inflammation and significant fibrosis. A similar pattern was observed in male patients, with a significantly higher rate of significant fibrosis in those with ALT between 30 and 50 U/L compared with those below 30 U/L (57.58% vs. 40.00%). Previous studies have reported that women with MASLD over 50 years of age are at higher risk of advanced fibrosis, possibly due to menopausal changes in fat distribution and declining estrogen levels [19,20,21,24,25]. However, in the present study, no significant association was found between sex or age and MASH or significant fibrosis. This may be because all enrolled patients had clinical indications for liver biopsy and therefore had relatively severe hepatic inflammation and fibrosis, which could attenuate differences related to age or sex. Larger studies are needed to verify these findings.

Lomonaco et al. [26] reported that an ALT threshold of 40 U/L failed to identify a significant number of diabetic patients with NAFLD and fibrosis. We reached a similar conclusion in our MASLD cohort, suggesting that lower ALT thresholds may be needed. Verma et al. [27] attempted to define an optimal ALT cutoff for predicting MASH or significant fibrosis but concluded that even a uniform limit of 35 U/L was insufficient. Building on these observations, we evaluated sex-specific lower ALT thresholds originally proposed for chronic hepatitis B in an MASLD population [11,12,13,14]. Our results suggest that lower ALT thresholds may increase the detection of MASH and significant fibrosis. However, the diagnostic performance of non-invasive markers [28,29,30,31,32,33] such as ALT [34] alone for assessing hepatic inflammation and fibrosis remains limited, with AUCs of 0.649 for MASH and 0.623 for significant fibrosis. Further prospective studies are needed, and current clinical practice should still combine non-invasive assessment tools or liver biopsy for accurate evaluation.

Type 2 diabetes mellitus, as an important metabolic disease, is closely associated with MASLD [35]. In the present study, the prevalence of type 2 diabetes mellitus was significantly higher in the normal-ALT group than in the elevated-ALT group (40.77% vs. 29.20%, *p* = 0.023). This is consistent with a previous study [36,37], which also found that among MASLD patients with type 2 diabetes mellitus and normal aminotransferase levels, 56% still had MASH. Therefore, in patients with type 2 diabetes mellitus or other metabolic disorders, MASH and significant fibrosis should be monitored closely even when ALT is normal. Further studies are needed to explore the relationship between type 2 diabetes mellitus and ALT thresholds, as well as the impact of type 2 diabetes mellitus on the prognosis of MASLD.

Our study has several limitations that must be acknowledged. First, its retrospective, two-center design is subject to the selection bias inherent to data derived from two tertiary referral hospitals. Because patients undergoing liver biopsy at these specialized centers typically present with clinical suspicion of advanced disease, the reported prevalence of MASH and fibrosis is likely inflated compared with that in the general MASLD population managed in community-based or primary care settings. To ensure consistency of multi-center data, all biochemical measurements in this study adopted the mutually recognized laboratory standards in Tianjin, and histological assessments were based on the interpretations of pathologists from the two centers, with a third pathologist consulted in cases of disagreement. Nevertheless, future prospective studies conducted in lower-tier healthcare settings are warranted to validate the generalizability of our findings to a broader, less-selected population.

Second, the sample size, though substantial, was relatively modest in subgroup analyses, which may limit the statistical power to detect certain associations. Third, the cross-sectional nature of the analysis precludes any assessment of causality or the predictive value of ALT thresholds for long-term hard outcomes such as cirrhosis or liver-related mortality [38,39]. Finally, our population was drawn from a Chinese cohort, and the generalizability of the proposed ALT thresholds to other ethnicities requires further investigation.

In conclusion, although all patients in this study had clinical indications for liver biopsy, the high prevalence of MASH and significant fibrosis among those with ALT within the conventional normal range was unexpected. After applying lower ALT thresholds, the rate of missed significant fibrosis was substantially reduced. Moreover, the lower ALT threshold was independently associated with MASH, regardless of sex and age. Therefore, clinicians should not rely solely on normal ALT to exclude the presence of hepatic inflammation and fibrosis. However, the independent diagnostic performance of ALT alone was limited, and ALT should still be combined with non-invasive assessment tools or liver biopsy in clinical practice.

## Figures and Tables

**Table 1:** Clinical characteristics of MASLD patients distinguished using diverse ALT ULN.

	ALT ≥ 50/40 (*n* = 274)	ALT < 50/40 (*n* = 130)	*p*
Age (years)	39 (29, 54)	51 (41, 58)	<0.001
Male, *n*(%)	154 (56.20)	53 (40.70)	0.004
BMI (kg/m^2^)	27.35 (25.00, 30.38)	27.00 (24.50, 30.00)	0.431
Type 2 diabetes mellitus, *n*(%)	80 (29.20)	53 (40.77)	0.023
CAP (dB/m)	299.00 (271.75, 328.00)	287.00 (257.00, 307.00)	<0.001
LSM (kPa)	7.70 (5.80, 10.70)	6.70 (4.73, 11.68)	0.078
AST (U/L)	57.60 (39.60, 88.25)	25.00 (19.00, 29.70)	<0.001
GGT (U/L)	78.50 (56.23, 130.00)	63.00 (35.00, 108.00)	0.003
ALB (g/L)	47.00 (43.75, 50.00)	45.00 (42.20, 48.00)	<0.001
PLT (×10^9^/L)	236.00 (187.75, 285.50)	211.00 (168.75, 270.25)	0.032
FBG (mmol/L)	5.80 (5.30, 6.50)	5.80 (5.20, 6.63)	0.993
TG (mmol/L)	1.78 (1.29, 2.39)	1.50 (1.22, 2.04)	0.004
LDL-C (mmol/L)	3.34 (2.76, 3.96)	3.20 (2.44, 3.68)	0.041
Ferritin (μg/L)	221.00 (126.50, 404.50)	129.00 (64.00, 205.00)	<0.001
Steatosis Score	2.33 ± 0.67	1.70 ± 0.59	<0.001
Lobular Inflammation Score	1.85 ± 0.47	1.51 ± 0.59	<0.001
Hepatocyte Ballooning Score	1.70 ± 0.46	1.43 ± 0.50	<0.001
MASH, *n*(%)	220 (80.20)	64 (49.23)	<0.001
F ≥ 2, *n*(%)	206 (75.10)	65 (50.00)	<0.001

Fibrosis stages ≥ F2 were defined as significant fibrosis.

**Table 2:** Comparison of general characteristics between patients with high-threshold versus low-threshold normal ALT (*n* = 130).

	Total with Conventionally Normal ALT (*n* = 130)	High-Threshold Normal Group (*n* = 89)	Low-Threshold Normal Group (*n* = 41)	*p*
Age (years)	51 (41, 58)	51 (42, 58)	51 (36, 60)	0.767
Male, *n* (%)	53 (40.77%)	33 (37.08%)	20 (48.78%)	0.143
BMI (kg/m^2^)	27.2 (24.6, 30.45)	27.65 (24.83, 30.73)	26.3 (24.35, 29.7)	0.179
Type 2 diabetes mellitus, *n*(%)	53 (40.77)	34 (38.20)	19 (46.34)	0.380
ALT (U/L)	30 (20, 38)	35 (28, 39)	18 (13.9, 23.2)	<0.001
GGT (U/L)	59.5 (36.75, 107.25)	69.1 (40, 107.5)	50.9 (24, 113.2)	0.096
PLT (10^9^/L)	218 (174, 268.5)	217 (175, 261.5)	219 (167.25, 272.75)	0.699
FBG (mmol/L)	5.8 (5.28, 6.73)	5.785 (5.3, 6.625)	5.85 (5.1, 7)	0.850
TG (mmol/L)	1.52 (1.23, 2.05)	1.57 (1.2325, 2.3125)	1.5 (1.22, 1.855)	0.483
LDL-C (mmol/L)	3.21 (2.45, 3.68)	2.98 (2.42, 3.64)	3.51 (2.65, 4.065)	0.059
Ferritin (U/L)	129.5 (64.25, 206.5)	182 (124.5, 269.5)	70 (35, 119)	<0.001
LSM (kPa)	6.9 (4.9, 11.375)	7.8 (5.35, 11.45)	6.2 (4.55, 11.65)	0.243
CAP (dB/m)	288 (256.5, 315)	292 (262.25, 319.75)	283 (251, 295)	0.043
APRI	0.31 (0.20, 0.51)	0.35 (0.25, 0.58)	0.19 (0.16, 0.31)	<0.001
FIB-4	1.1 (0.80, 1.90)	1.24 (0.81, 1.89)	0.97 (0.76, 2.01)	0.405
MASH, *n* (%)	64 (49.23%)	48 (53.93%)	16 (39.02%)	0.082
Fibrosis, *n* (%)				
F0	40 (30.77%)	23 (25.84%)	17 (41.46%)	
F1	25 (19.23%)	16 (17.98%)	9 (21.95%)	
F ≥ 2	65 (50.00%)	50 (56.18%)	15 (36.59%)	0.002

Total with conventionally normal ALT was defined as <50 for males and <40 for females. The high-threshold normal group was defined as 30–50 U/L for males and 19–40 U/L for females. The low-threshold normal group was defined as <30 U/L for males and <19 U/L for females. Fibrosis stages ≥ F2 were defined as significant fibrosis.

**Table 3:** General characteristics of subjects by MASH or significant fibrosis.

	MASH or Significant Fibrosis (*n* = 80)	Without MASH or Significant Fibrosis (*n* = 50)	*p*
Age (years)	51.5 (43.25, 60)	49 (35, 54.25)	0.023
Male, *n* (%)	32 (48)	21 (29)	0.482
BMI (kg/m^2^)	27.8 (25.48, 30.8)	26.45 (23.48, 29.23)	0.022
Type 2 diabetes mellitus, *n* (%)	37 (46.25)	16 (32.00)	0.108
ALT (U/L)	31.85 (24.63, 37.93)	24.1 (19, 37.25)	0.029
GGT (U/L)	61 (38, 104.05)	58.5 (28.75, 117.25)	0.918
PLT (10^9^/L)	199 (161, 245)	248 (199.5, 292.5)	0.002
FBG (mmol/L)	6.1 (5.5, 7.475)	5.5 (5.05, 6.3)	<0.001
TG (mmol/L)	1.51 (1.2, 2.05)	1.52 (1.25, 2.02)	0.563
LDL-C (mmol/L)	3.16 (2.425, 3.67)	3.3 (2.47, 3.73)	0.193
Ferritin (U/L)	149 (61.5, 238)	123 (75, 202)	0.536
LSM (kPa)	9.2 (6.1, 15.1)	5.5 (4.5, 7.5)	0.001
CAP (dB/m)	292 (261, 330)	282 (250, 298)	0.043
APRI	0.36 (0.26, 0.68)	0.20 (0.17, 0.34)	<0.001
FIB-4	1.672 (0.94, 2.334)	0.88 (0.51, 1.17)	0.001

Total with conventionally normal ALT was defined as <50 for males and <40 for females. Fibrosis stages ≥ F2 were defined as significant fibrosis.

**Table 4:** Prevalence of MASH and liver fibrosis stratified by age and lower ALT ULN in MASLD patients with conventionally normal ALT.

	Age > 50 (*n* = 68)		*p*	Age ≤ 50 (*n* = 62)		*p*
	High-Threshold Normal Group (*n* = 46)	Low-Threshold Normal Group (*n* = 22)		High-Threshold Normal Group (*n* = 43)	Low-Threshold Normal Group (*n* = 19)	
MASH, *n* (%)	25 (54.35%)	9 (40.91%)	0.219	23 (53.49%)	7 (36.84%)	0.175
Fibrosis, *n* (%)						
F0	12 (26.09%)	7 (31.8%)		11 (25.58%)	10 (52.63%)	
F1	9 (19.57%)	4 (18.21%)		7 (16.28%)	5 (26.32%)	
F ≥ 2	25 (54.35%)	11 (50%)	0.121	25 (58.14%)	4 (21.05%)	0.144

Total with conventionally normal ALT was defined as <50 for males and <40 for females. The high-threshold normal group was defined as 30–50 U/L for males and 19–40 U/L for females. The low-threshold normal group was defined as <30 U/L for males and <19 U/L for females. Fibrosis stages ≥ F2 were defined as significant fibrosis.

**Table 5:** Prevalence of MASH and liver fibrosis stratified by gender and lower ALT ULN in MASLD patients with conventionally normal ALT.

	Male (*n* = 53)		*p*	Female (*n* = 77)		*p*
	High-Threshold Normal Group (*n* = 33)	Low-Threshold Normal Group (*n* = 20)		High-Threshold Normal Group (*n* = 56)	Low-Threshold Normal Group (*n* = 21)	
MASH, *n* (%)	14 (42.42%)	10 (50.00%)	0.776	34 (60.71%)	6 (28.57%)	0.001
Fibrosis, *n* (%)						
F0	8 (24.24%)	8 (40.00%)		15 (26.79%)	9 (42.86%)	
F1	6 (18.18%)	4 (20.00%)		10 (17.86%)	5 (23.81%)	
F ≥ 2	19 (57.58%)	8 (40.00%)	0.030	31 (55.36%)	7 (33.33%)	0.021

Total with conventionally normal ALT was defined as <50 for males and <40 for females. The high-threshold normal group was defined as 30–50 U/L for males and 19–40 U/L for females. The low-threshold normal group was defined as <30 U/L for males and <19 U/L for females. Fibrosis stages ≥ F2 were defined as significant fibrosis.

**Table 6:** Multivariate analysis of risk factors associated with MASH or significant fibrosis in patients with conventionally normal ALT.

	MASH		F ≥ 2	
Variables	*OR* (95% *CI*)	*p*	*OR* (95% *CI*)	*p*
Age				
>50	1.743 (0.549, 5.530)		1.043 (0.391, 2.780)	
≤50	1.294 (0.729, 2.297)	0.378	1.006 (0.377, 2.682)	0.933
Sex				
Male	0.665 (0.369, 1.199)		1.946 (0.608, 6.225)	
Female	1.743 (0.549, 5.530)	0.346	0.529 (0.380, 2.824)	0.284
Lower ALT ULN				
=30/19–50/40 U/L	5.188 (1.494, 18.018)		1.725 (0.530, 5.615)	
<30/19 U/L	0.086 (0.033, 0.255)	<0.001	0.570 (0.176, 1.848)	0.365

Fibrosis stages ≥ F2 were defined as significant fibrosis.

## Data Availability

The data presented in this study are available on request from the corresponding author. The data are not publicly available due to privacy and ethical restrictions.

## Supplementary Materials

The following supporting information can be downloaded at https://www.mdpi.com/article/10.3390/jcm15187212/s1: Figure S1: ROC curves for the detection of (A) MASH using ALT, CAP, and FBG, and (B) significant fibrosis using ALT, LSM, FIB-4, and APRI in MASLD patients with conventionally normal ALT.
